# Rapid Response Protocol for Large-Bore Access Thrombosis After Percutaneous LVAD Removal

**DOI:** 10.1016/j.jaccas.2025.105613

**Published:** 2025-10-07

**Authors:** Whitney M. Graham, Leann M. Nugent, Ahmad A. Awan, Edward C. Bergen

**Affiliations:** a Cardiac Catheterization Laboratory, Lake Charles Memorial Hospital, Lake Charles, Louisiana, USA; b Heart & Vascular Center, Lake Charles Memorial Hospital, Lake Charles, Louisiana, USA

**Keywords:** cardiogenic shock, large-bore device, limb ischemia, mechanical circulatory support, mechanical thrombectomy, vascular thrombosis

## Abstract

**Background:**

Vascular thrombosis after large-bore device removal is a rare but critical complication that can result in limb ischemia or the need for open surgical intervention. Rapid identification and endovascular management are key to improving patient outcomes.

**Project Rationale:**

With the 2025 American Heart Association/American College of Cardiology guidelines assigning a Class IIa recommendation for mechanical circulatory support in patients experiencing ST-segment elevation myocardial infarction with cardiogenic shock, the use of percutaneous left ventricular assist devices is increasing. However, protocols for safe closure after removal remain limited, prompting the need for structured quality improvement process managing.

**Project Summary:**

A patient required support from a percutaneous left ventricular assist device for 5 days. During removal, angiography revealed extensive iliac artery thrombosis. Multidisciplinary response included contralateral imaging, mechanical thrombectomy with dedicated large-vessel system, and subsequent closure with a collagen-based vascular closure device.

**Take-Home Message:**

Routine angiography after large-bore device removal enables early complication detection. Mechanical thrombectomy provides an effective, minimally invasive option to avoid surgical escalation.

## Graphical Abstract

**Figure:**
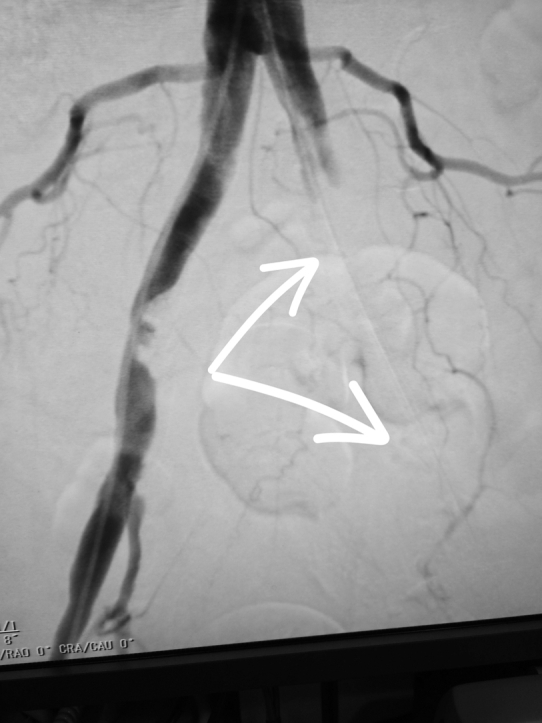

Vascular complications after large-bore device removal represent high-stakes challenges. A rare but critical complication that can result is limb ischemia or the need for open surgical intervention. Rapid identification and endovascular management are key to improve patient outcomes and minimize procedural morbidity. With the 2025 American Heart Association/American College of Cardiology guidelines now assigning a Class IIa recommendation for mechanical circulatory support (MCS) in selected ST-segment elevation myocardial infarction (STEMI) patients with cardiogenic shock,^1^ the use of percutaneous left ventricular assist device (pLVAD) support, such as the Impella CP (Abiomed), is increasingly relevant in high-acuity settings.^2^, ^3^, ^4^, ^5^ The guidelines also downgraded the use of routine intra-aortic balloon pumps and extracorporeal membrane oxygenation.^3^ This shift underscores the growing use of pLVAD in high-acuity cases. Although protocols exist for insertion and support, fewer standardized pathways exist for addressing complications related to large-bore access site closure.

**Take-Home Messages**

• Routine angiography after large-bore MCS removal ensures early detection of vascular complications.
• Multidisciplinary collaboration and device-based thrombectomy can obviate the need for open surgical intervention.
• Updated protocols serve as a model for institutions managing high-risk MCS patients.

## Case Summary Prompting the Project Launch

A 57-year-old male with prior cardiac history presented with cardiogenic shock after an acute myocardial infarction. MCS was initiated using a pLVAD.^2^^,^^4^ The support device remained in place for 5 days. At the time of planned removal, the patient remained hemodynamically tenuous with persistently reduced cardiac function, though stable enough to proceed. The pLVAD was removed, and a measuring system for a collagen-based vascular closure device was inserted to determine deployment depth. Minimal to no blood flow was noted from 2 successive depth measurements, which indicated possible acute limb ischemia complication.

**Figure 1:**
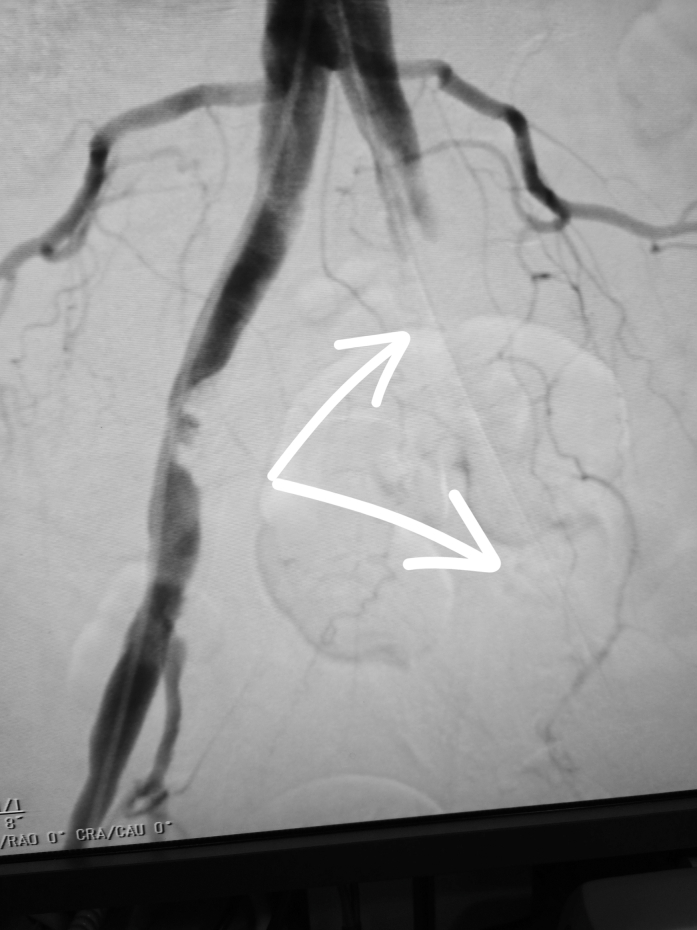
Digital Subtraction Angiography Performed After pLVAD Removal The arrows indicate extensive occlusive thrombus extending from the left common iliac artery to the left external iliac artery.

A 14-F introducer sheath was inserted to maintain arterial access, and contralateral femoral access was obtained to perform angiography. Imaging revealed extensive thrombus extending from the common iliac to external iliac artery at the site of previous pLVAD insertion (Figure 1).^3^^,^^5^ An urgent multidisciplinary discussion was held with the cardiovascular surgeon and interventional cardiologists. The decision was made to proceed with large-vessel mechanical thrombectomy using the Pounce thrombectomy system (Surmodics), which uses a nitinol wire and self-expanding funnel system to capture and remove thrombus in large-caliber vessels.^6^ Three passes were performed per protocol, successfully retrieving the clot burden. Post-thrombectomy imaging confirmed restored flow from the common iliac through the superficial femoral artery.

After successful thrombectomy, the closure measuring system was reinserted to confirm vessel depth. Subsequently, the Manta (Teleflex), a collagen-based vascular closure device, was successfully deployed. Final angiography through contralateral access demonstrated normal blood flow and hemostasis at arteriotomy.

## Project Rationale

This case highlighted a rare but critical complication of large-bore device removal: access site thrombosis. American Heart Association/American College of Cardiology guidelines regarding STEMI now recommend microaxial flow pumps (mAFPs) as the preferred MCS strategy in selected STEMI-related cardiogenic shock patients,^2^ increasing the likelihood that clinicians will encounter these access-related complications. Standardized pathways for device insertion and support exist, but fewer structured protocols are available for managing complications after removal.^3^

## Project Description

**Figure 2:**
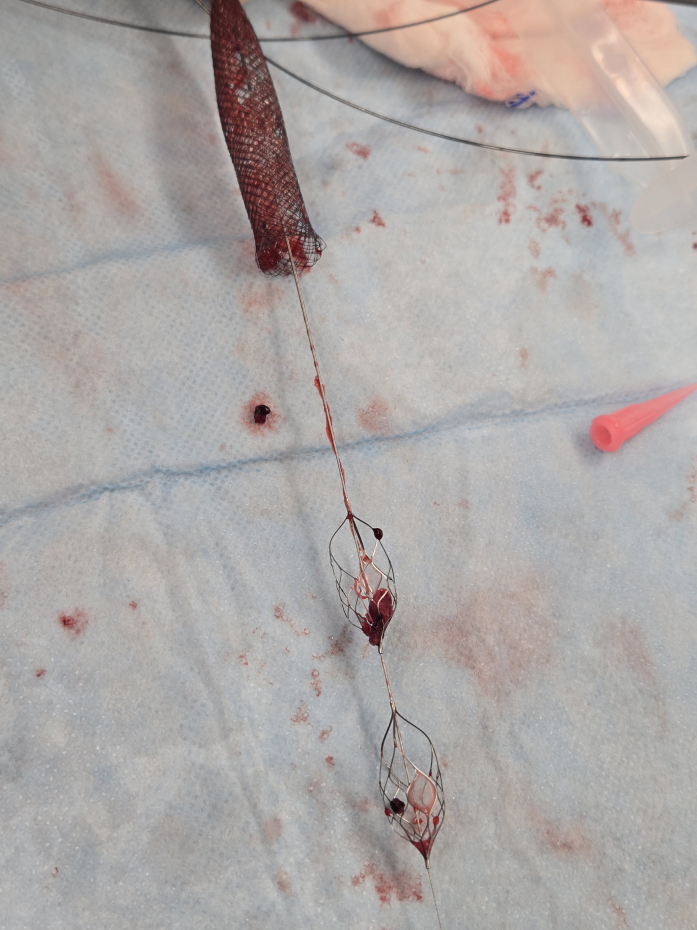
The Pounce Thrombectomy System Shown are the self-expanding funnel and nitinol basket wire demonstrating thrombus removed.

**Figure 3:**
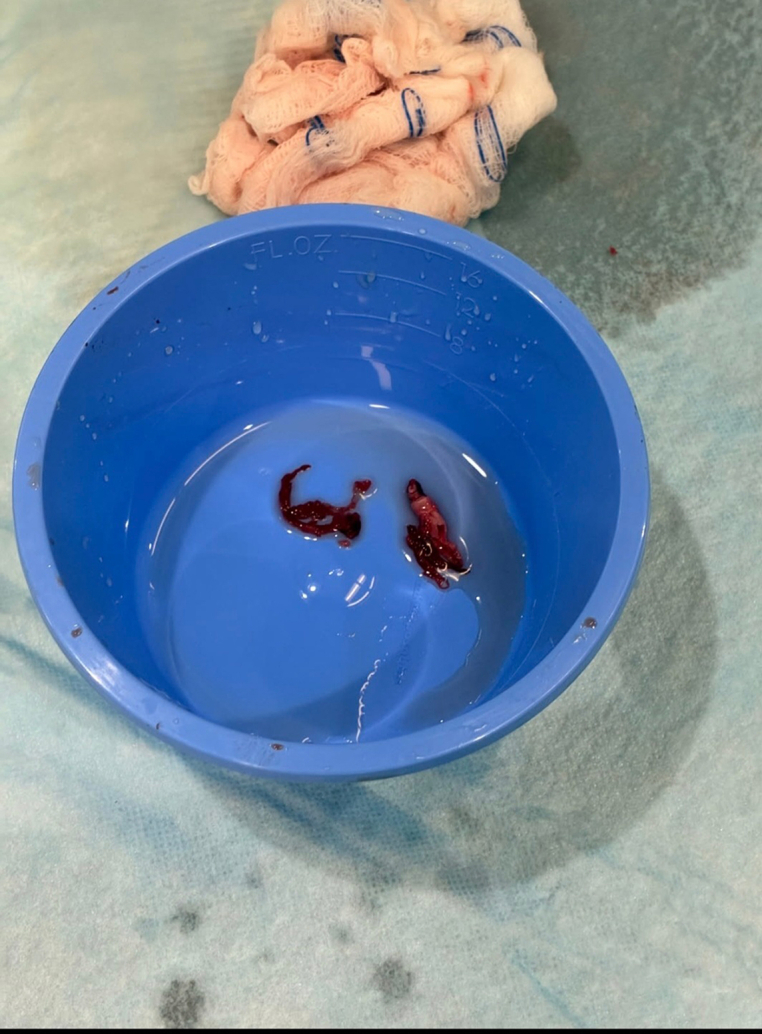
Retrieved Thrombus From the Left Common Iliac and Left External Iliac Arteries

**Figure 4:**
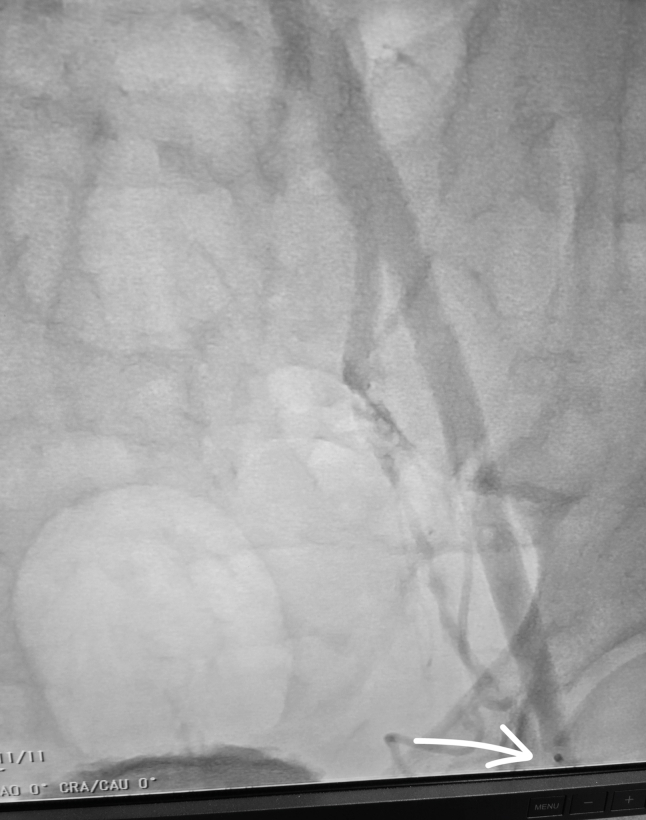
Final Angiogram Via Contralateral Access After Closure Angiography confirms restored arterial patency from the left common iliac artery to the left common femoral artery with hemostasis achieved at access site. The arrow denotes the stainless-steel suture lock in place.

The pLVAD device was removed, and a 14-F introducer sheath was placed to maintain access. Imaging confirmed thrombus throughout the iliac system. A multidisciplinary team including interventional cardiology and cardiovascular surgery elected to use a dedicated large-vessel mechanical thrombectomy device. Thrombus was retrieved, resulting in restoration of flow (Figures 2 and 3). Subsequent arteriotomy closure was successfully achieved with a collagen-based closure device (Figure 4).^5^

## Project Deliverables

**Figure 5:**
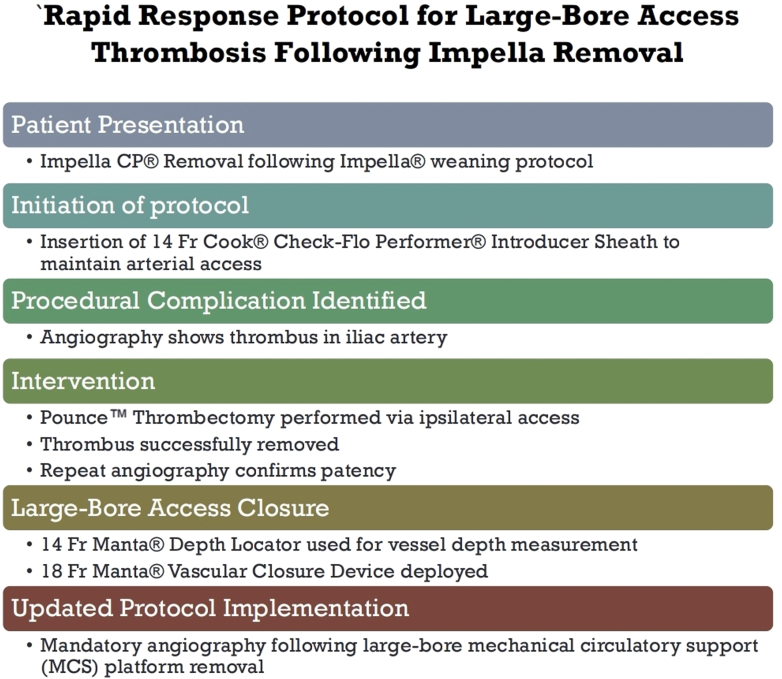
Flowchart Demonstrating the Events Leading to the Proposed Protocol Update

After this event, institutional protocols were updated to require routine angiography after mAFP removal, either by maintaining ipsilateral sheath access or by obtaining contralateral femoral access. This change is intended to ensure rapid recognition of access-related complications and reduce delays to intervention (Figure 5).^3^^,^^6^

## Project Outcome, Impact, and Future Directions

The intervention avoided open surgical thrombectomy and limb ischemia. Quantitatively, time from detection to revascularization was 45 minutes. Future plans include prospective tracking of outcomes such as ischemic complications, need for surgical conversion, and cost impact compared with historical practice.

## Discussion

This case demonstrates how rapid identification of vascular thrombosis and use of mechanical thrombectomy can effectively resolve large-bore access complications. A multidisciplinary, image-guided approach avoided escalation to surgery in an already compromised patient, aligning with evolving guideline-directed care.^5^ Published evidence supports both the growing role of mAFPs^2^^,^^4^ and the safety of large-bore closure techniques.^3^^,^^5^ This project establishes a reproducible framework that may be adopted by other institutions.

## Conclusions

Rapid identification, multidisciplinary collaboration, and mechanical thrombectomy successfully salvaged a limb-threatening complication of MCS removal. The protocol update requiring routine angiography after large-bore device removal enhances patient safety and standardizes care for future cases.

## Funding Support and Author Disclosures

The authors have reported that they have no relationships relevant to the contents of this paper to disclose.
